# Environmental Biodegradation of Water-Soluble Polymers: Key Considerations and Ways Forward

**DOI:** 10.1021/acs.accounts.2c00232

**Published:** 2022-08-05

**Authors:** Michael Zumstein, Glauco Battagliarin, Andreas Kuenkel, Michael Sander

**Affiliations:** † Division of Environmental Geosciences, Centre for Microbiology and Environmental Systems Science, University of Vienna, Josef-Holaubek-Platz 2, 1090 Vienna, Austria; ‡ BASF SE, Carl-Bosch-Strasse 38, 67056 Ludwigshafen am Rhein, Germany; § Institute of Biogeochemistry and Pollutant Dynamics, Department of Environmental Systems Science, ETH Zurich, Universitätstrasse 16, 8092 Zurich, Switzerland

## Abstract

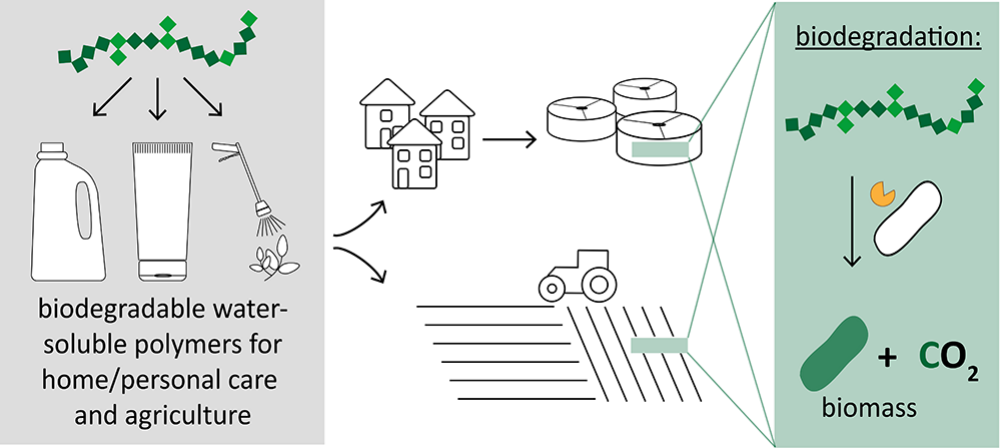

Water-soluble polymers (WSPs) have unique properties that are valuable in diverse applications ranging from home and personal care products to agricultural formulations. For applications that result in the release of WSPs into natural environments or engineered systems, such as agricultural soils and wastewater streams, biodegradable as opposed to nonbiodegradable WSPs have the advantage of breaking down and, thereby, eliminating the risk of persistence and accumulation. In this Commentary, we emphasize central steps in WSP biodegradation, discuss how these steps depend on both WSP properties and characteristics of the receiving environment, and highlight critical requirements for testing WSP biodegradability.

## Water-Soluble Polymers (WSPs): Use Areas and Entry Pathways to the Environment

WSPs are chemically diverse, ranging from linear uncharged homopolymers (e.g., poly(ethylene oxide)s) to branched and charged copolymers (e.g., polyacrylates, modified polysaccharides, and polyamino acids).^1^ Because of their chemical diversity, WSPs cover a wide range of properties and functionalities (e.g., thickening, stabilization, and emulsification) that are critical to their use in numerous applications. The use of WSPs in some applications results in the release of WSPs into engineered and natural environments. For example, WSPs in home and personal care products enter wastewater streams and sewage treatment plants. Similarly, WSPs used in agrochemical formulations can enter agricultural soils.^2^ Despite the known entry pathways and high usage, little attention is currently paid to the environmental fate of WSPs.^3^ In this context, replacing nonbiodegradable with biodegradable WSPs offers a unique benefit: biodegradable WSPs undergo breakdown to defined metabolic end products.^2,4,5^ In this Commentary, we highlight key concepts on the biodegradation of WSPs in natural and engineered environments and discuss experimental approaches to test WSP biodegradation as well as challenges associated with these approaches. The environmental fate of nonbiodegradable WSPs is beyond the scope of this commentary.^1^

## WSP Biodegradation and Key Influencing Factors

**Figure 1:**
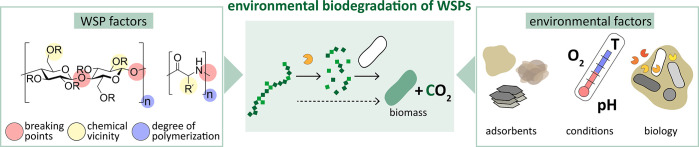
Schematic depiction of the environmental biodegradation of water-soluble polymers (WSPs) and key factors governing this process. WSP factors: the presence of a bond in the backbone that is susceptible to cleavage in the receiving environment (glycosidic and peptide bonds shown as examples), chemical vicinity of the breaking point (e.g., variation in the chemistry of the repeating units, substitutions, and stereochemistry), and degree of polymerization. Environmental factors: types and amounts of adsorbents (e.g., mineral phases, organic matter, and extracellular polymeric substances), abiotic conditions (e.g., redox, temperature, and pH), and abundance and activity of degrading organisms and enzymes. The process depiction of biodegradation (middle) focuses on carbon. R = H or a substitution such as CH_2_COOH or CH_2_CH_2_OH; R′ = amino acid side chain.

WSP biodegradation is the process in which all components of a WSP are completely metabolically utilized by organisms in the receiving environment (Figure 1). For carbon-based WSPs, biodegradation describes the conversion of polymer carbon to the metabolic end products CO_2_ and biomass under aerobic conditions (and potentially also CH_4_ under anaerobic conditions).^6^ Demonstrating the dissipation of a WSP (without demonstrating the conversion to defined end products) is insufficient for claiming its biodegradation. In regulatory and product certification contexts, this definition needs to be complemented by a time period over which an explicit, minimal extent of biodegradation needs to be attained (which can be application specific) and by specifying the environment in which biodegradation is assessed. Importantly, while biodegradation is a desired trait of some WSPs, the properties of a WSP that bestow biodegradation ideally do not compromise the functionality and performance of the WSP during its use period.

To inform the design and the regulation of biodegradable WSPs, the rates and extents of biodegradation need to be studied and the factors influencing these parameters need to be understood (Figure 1). In the following paragraphs, we discuss known and anticipated key factors. Importantly, WSP biodegradability is a function of both polymer chemistry and the characteristics of the receiving environment.

As opposed to small organic molecules, the large molecular weight of most WSPs impairs their direct uptake into microbial cells, which is required for intracellular metabolic utilization to the above-defined biodegradation end products.^7,8^ Consequently, the initial, and presumably often rate-limiting, step in the biodegradation of WSPs is the extracellular breakdown of the WSP that results in products sufficiently small for cellular uptake. WSP biodegradation is expected to slow down with increasing WSP chain length because more cleavages are needed to form sufficiently small products. However, systematic studies on the effect of WSP molecular weight on biodegradation rates are missing. The breakdown of the WSP can either be catalyzed by enzymes or occur abiotically and typically involves hydrolytic (e.g., in the case of polyamino acids or polysaccharides) or, less commonly, oxidative reactions (e.g., in the case of poly(ethylene glycol)). It is conceivable that some WSPs, unlike structural polymers, may be directly taken up by microorganism, as previously described for alginate uptake by a *Sphingomonas* strain.^9^ Biodegradation rates of such WSPs may be less dependent on extracellular breakdown.

The breakdown of WSPs necessitates the presence of labile chemical bonds in the WSP backbone that are susceptible to cleavage reactions. Many biomacromolecules, including polysaccharides and polypeptides, contain such “intended breaking points” (i.e., glycosidic and peptide bonds), and biomacromolecules thus offer structural motifs for biodegradable WSPs.^10,11^ However, cleavage rates of bonds of the same class can vary depending on the chemistry and steric factors of neighboring functional groups, particularly if the cleavage is catalyzed by enzymes requiring a specific conformation of the backbone in their active site. Examples that can modify rates of bond cleavage include the side-chain (stereo)chemistry of polyamino acids and the type and degree of substitution of polysaccharides. Importantly, side-chain chemistry, stereochemistry, and the type and degree of substitution may not be uniform along the WSP chain, giving rise to variable rates of backbone cleavage and thus intramolecular variations in the biodegradability of a WSP.

Additional variability in degradation arises from WSP chains being highly flexible (in contrast to chains in structural polymers), allowing for the WSP to adopt different conformations depending on the environmental conditions such as solution pH and ionic composition. These conformations may have different susceptibilities to enzymatic breakdown. Furthermore, WSPs may adsorb to environmental surfaces (e.g., mineral phases and organic matter in soils or extracellular polymeric substances in wastewater), a process that generally decreases the availability of the WSP to degrade enzymes and microbial cells. Adsorption may slow down biodegradation, particularly for WSPs that require breakdown by enzymes in solution.^12^ Preferential adsorption of specific segments of a WSP chain may result in slower biodegradation of these segments.

Among the environmental factors that control the biodegradation of WSPs are the abundance and activity of enzymes and organisms that are capable of breaking down and metabolically utilizing WSPs as well as abiotic factors such as temperature (which affects the enzymatic activity), water content (e.g., in soils), nutrient availability, solution pH (which determines the protonation and charge state of ionizable WSPs, enzymes, and adsorbents and thus governs polymer–sorbent electrostatic interactions), and redox conditions (which control the rates and pathways of intracellular metabolic processing).^13^

## Testing WSP Biodegradation

Rigorous experimental testing is needed to establish, certify, and register a WSP as biodegradable and to thereby ensure that the WSP will not persist in the environment. This testing must involve demonstrating that the WSP is converted to the above-defined biodegradation end products.^14^ Beyond demonstrating biodegradation in a targeted format for regulatory purposes, there is a need for systematic studies that reveal generalizable principles on WSP biodegradation. In the following section, we discuss promising experimental and analytical approaches to study WSP biodegradation, and we highlight associated challenges. Notably, these approaches and challenges are independent of the feedstock of the WSP and thus equally apply to synthetic, fossil-based WSPs and to WSPs derived from natural biopolymers.

The most direct approach to demonstrate WSP biodegradation is the use of laboratory experiments in which a WSP is incubated in the desired medium (e.g., soil or wastewater) under conditions representative of the respective receiving environment and in which respirometric analysis is used to quantify the amount of formed CO_2_ (or CO_2_ and CH_4_ under methanogenic conditions).^15^ In aerobic incubation experiments, CO_2_ formation measurements can be complemented with measurements of O_2_ consumption. However, processes other than WSP biodegradation may lead to O_2_ consumption (e.g., nitrification) and need to be considered and, if needed, controlled for.

Guidelines for testing the biodegradation of small molecules (e.g., OECD 301 B and F, OECD 310) as well as methods for testing the biodegradation of structural polymers (e.g., ISO 17556 and ISO 19679) in defined environmental compartments may serve as a useful starting point for testing WSP biodegradation. However, the applicability of existing methods to WSPs needs to be critically assessed, and if needed, methods require WSP-specific adaptations. For example, biodegradation tests for small molecules commonly use a microbial inoculum from the targeted environment (e.g., the aeration tank of a wastewater treatment plant). This inoculum is typically diluted and aerated to decrease the amount of natural substrate prior to the incubation experiment. Such treatments likely remove most or all extracellular enzymes from the inoculum. When used in biodegradation experiments of WSPs that require breakdown by extracellular enzymes, such treatments may result in artificially low biodegradation rates. Another aspect that warrants careful consideration is the temperature at which biodegradation tests are conducted. For example, conducting tests at the annual mean temperature of a certain environment might not adequately capture biodegradation rates in these systems if biodegradation rates do not scale linearly with temperature. Systematic studies on the temperature dependence of WSP biodegradation are needed to inform the selection of adequate testing temperatures and to critically assess the use of experimental biodegradation rates at higher temperatures to predict biodegradation rates at lower, environmentally relevant temperatures. Finally, extrapolating biodegradation extents over time from experiments in which small biodegradation extents were measured needs to be approached carefully given the above-mentioned multitude of factors that can lead to variable rates of biodegradation along the WSP chain (i.e., nonuniform chemistry along the WSP chain, conformational changes in WSPs in response to changes in environmental conditions, and adsorption of WSPs to environmental surfaces).

Biodegradation tests benefit from including substrates known to biodegrade in the respective environment.^16^ First, the use of such substrates ensures the proper operation of the testing systems and confirms biological activity. Second, such substrates may also be used as references to compare biodegradation across WSPs and environments. Although frequently used reference substrates such as glucose and cellulose serve the first purpose, they may biodegrade so readily across systems that they cannot help identify the system factors that control WSP biodegradation. A careful selection of reference substrates is particularly warranted if the conversion extent of WSP carbon to CO_2_ is reported relative to the conversion extent of the reference substrate carbon to CO_2_. Such normalization stipulates that the fraction of carbon that is converted to CO_2_ vs incorporated into biomass is similar for WSP and the reference substrate.

Carbon isotope labeling of WSPs provides the unique opportunity to directly track their conversion to biodegradation end products CO_2_ (and CH_4_) and microbial biomass at high sensitivity and selectivity. Such labeling, particularly with the radioisotope ^14^C, has commonly been used to study the biodegradation of small organic molecules. There also is precedence for using ^13^C labeling to study the biodegradation of structural polymers.^6^ However, isotopically labeled monomers or biomolecules used as building blocks in WSPs are expensive (if available), which restricts the syntheses of labeled WSPs to small scales. Such small-scale syntheses may result in WSPs with properties differing from those produced on a larger, industrial scale. Therefore, we consider isotope-labeling approaches not generally suited for broad and general testing of WSP biodegradation but rather for detailed investigations of specific polymers and receiving environments. Examples are the elucidation of the biodegradation of a specific part of a WSP or of a biotransformation intermediate.

Respirometric analyses are practically restricted to laboratory incubations and cannot readily be used for in situ tests in receiving environments. The latter would, however, be possible through analytical techniques that allow the quantification of the decrease in the concentration of a WSP during its biodegradation. Approaches to analyze WSPs based on liquid- and size-exclusion chromatography coupled to mass spectrometry were recently presented for PEG^17,18^ but await the demonstration of their applicability to other WSPs. This demonstration may prove difficult for WSPs with different chemistries (e.g., charged polyelectrolytes as opposed to uncharged PEG). A major challenge in quantifying WSPs in environmental samples is the development of protocols that enable exhaustive extraction of the WSP prior to quantification.^16^ Additionally, the polydispersity of most WSPs, in combination with the typically low concentrations under realistic scenarios, requires analytical techniques to be highly sensitive. Once developed, however, such methods would open new possibilities to obtain insights into biodegradation pathways and to study the effect of WSP adsorption on WSP biodegradation.^19^

Because WSP biodegradation experiments are time- and labor-intensive, there is a need for automation and miniaturization of testing setups as well as for establishing scientifically sound methods for the fast preliminary screening of candidate WSPs. Furthermore, experimental studies ought to be complemented with modeling efforts. Integrative statistical analyses of the data generated in biodegradation testing, models based on the parametrization of kinetics and the pathways of WSP biodegradation, and in-depth analyses of physicochemical parameters of WSPs are prerequisites to advancing our capability to predict WSP biodegradation and guide the design of biodegradable WSPs.

The development and testing of biodegradable WSPs can leverage the previously generated knowledge on the biodegradation of small molecules and structural polymers. Akin to small molecules, WSPs are prone to adsorption to particle surfaces in the environment, calling for a consideration of the effect of adsorption on biodegradation. Contrary to small molecules, however, WSPs can adopt different conformations in response to changes in solution chemistry. Furthermore, because the chemistry can vary along the chain of a WSP, WSPs likely show more complex biodegradation dynamics than low-molecular-weight molecules. Akin to biodegradable structural polymers, biodegradable WSPs commonly require extracellular breakdown for biodegradation to occur. Contrary to structural polymers, however, WSPs do not possess a solid surface that on one side can be colonized by degrading microorganisms and to which enzymes can bind and on the other side can limit the availability of the bulk material to biotic degradation. Biodegradation tests of WSPs need to be both scientifically rigorous and highly practical. We acknowledge that biodegradability is but one desired property of WSPs that ought not to interfere with other warranted properties of the WSP during use and production (e.g., functionality, stability, nontoxicity, and sustainable feedstock sourcing).^1^ An interdisciplinary approach with expertise from polymer chemistry, environmental chemistry, microbiology, and environmental engineering, among other fields, is therefore needed for the development of biodegradable WSPs.

## References

[ref1] Vandermeulen G. W. M.; Boarino A.; Klok H.-A. Biodegradation of Water-Soluble and Water-Dispersible Polymers for Agricultural, Consumer, and Industrial Applications—Challenges and Opportunities for Sustainable Materials Solutions. J. Polym. Sci. 2022, 60, 1797–1813. 10.1002/pol.20210922.

[ref2] Polymers in Liquid Formulations (PLFs); Royal Society of Chemistry. https://www.rsc.org/new-perspectives/sustainability/polymers-in-liquid-formulations-plfs/ (accessed 06-08-2021).

[ref3] Arp H. P. H.; Knutsen H. Could We Spare a Moment of the Spotlight for Persistent, Water-Soluble Polymers. Environ. Sci. Technol. 2020, 54, 3–5. 10.1021/acs.est.9b07089. 31845804

[ref4] Künkel A.; Becker J.; Börger L.; Hamprecht J.; Koltzenburg S.; Loos R.; Schick M. B.; Schlegel K.; Sinkel C.; Skupin G.; Yamamoto M. Polymers, Biodegradable. Ullmann’s Encyclopedia of Industrial Chemistry; Wiley-VCH Verlag GmbH & Co. KGaA: Weinheim, Germany, 2016; pp 1231–1259 10.1002/14356007.n21_n01.pub2.

[ref5] Kümmerer K.; Dionysiou D. D.; Olsson O.; Fatta-Kassinos D. A Path to Clean Water. Science 2018, 361, 222–224. 10.1126/science.aau2405. 30026210

[ref6] Zumstein M. T.; Schintlmeister A.; Nelson T. F.; Baumgartner R.; Woebken D.; Wagner M.; Kohler H.-P. E.; McNeill K.; Sander M. Biodegradation of Synthetic Polymers in Soils: Tracking Carbon into CO _2_ and Microbial Biomass. Sci. Adv. 2018, 4, 1–8. 10.1126/sciadv.aas9024. PMC6059733 30050987

[ref7] Nikaido H.; Vaara M. Molecular Basis of Bacterial Outer Membrane Permeability. Microbiol Rev. 1985, 49, 1–32. 10.1128/mr.49.1.1-32.1985. 2580220 PMC373015

[ref8] Pantoja S.; Lee C. Peptide Decomposition by Extracellular Hydrolysis in Coastal Seawater and Salt Marsh Sediment. Marine Chemistry 1999, 63, 273–291. 10.1016/S0304-4203(98)00067-X.

[ref9] Hisano T.; Yonemoto Y.; Yamashita T.; Fukuda Y.; Kimura A.; Murata K. Direct Uptake of Alginate Molecules through a Pit on the Bacterial Cell Surface: A Novel Mechanism for the Uptake of Macromolecules. Journal of Fermentation and Bioengineering 1995, 79, 538–544. 10.1016/0922-338X(95)94744-C.

[ref10] Obst M.; Steinbüchel A. Microbial Degradation of Poly(Amino Acid)s. Biomacromolecules 2004, 5, 1166–1176. 10.1021/bm049949u. 15244426

[ref11] Seebach D.; Gardiner J. β-Peptidic Peptidomimetics. Acc. Chem. Res. 2008, 41, 1366–1375. 10.1021/ar700263g. 18578513

[ref12] Cai P.; Huang Q.-Y.; Zhang X.-W. Interactions of DNA with Clay Minerals and Soil Colloidal Particles and Protection against Degradation by DNase. Environ. Sci. Technol. 2006, 40, 2971–2976. 10.1021/es0522985. 16719099

[ref13] Zumstein M. T.; Werner J. J.; Helbling D. E. Exploring the Specificity of Extracellular Wastewater Peptidases to Improve the Design of Sustainable Peptide-Based Antibiotics. Environ. Sci. Technol. 2020, 54, 11201–11209. 10.1021/acs.est.0c02564. 32790288

[ref14] Albertsson A.-C.; Hakkarainen M. Designed to Degrade. Science 2017, 358, 872–873. 10.1126/science.aap8115. 29146799

[ref15] Zumstein M. T.; Narayan R.; Kohler H.-P. E.; McNeill K.; Sander M. Dos and Do Nots When Assessing the Biodegradation of Plastics. Environ. Sci. Technol. 2019, 53, 9967–9969. 10.1021/acs.est.9b04513. 31418543

[ref16] Albright V. C.; Chai Y. Knowledge Gaps in Polymer Biodegradation Research. Environ. Sci. Technol. 2021, 55, 11476–11488. 10.1021/acs.est.1c00994. 34374525

[ref17] Mairinger T.; Loos M.; Hollender J. Characterization of Water-Soluble Synthetic Polymeric Substances in Wastewater Using LC-HRMS/MS. Water Res. 2021, 190, 116745 10.1016/j.watres.2020.116745. 33360422

[ref18] Huppertsberg S.; Zahn D.; Pauelsen F.; Reemtsma T.; Knepper T. P. Making Waves: Water-Soluble Polymers in the Aquatic Environment: An Overlooked Class of Synthetic Polymers. Water Res. 2020, 181, 115931 10.1016/j.watres.2020.115931. 32505887

[ref19] Haider T. P.; Völker C.; Kramm J.; Landfester K.; Wurm F. R. Plastics of the Future? The Impact of Biodegradable Polymers on the Environment and on Society. Angew. Chem., Int. Ed. 2019, 58, 50–62. 10.1002/anie.201805766. 29972726

